# Understanding the effects of opioids vs non-opioids in the treatment of neonatal abstinence syndrome, an in vitro model

**DOI:** 10.3389/fped.2022.1068330

**Published:** 2022-11-22

**Authors:** Thitinart Sithisarn, Sandra J. Legan, Philip M. Westgate, Henrietta S. Bada, Melinda E. Wilson

**Affiliations:** ^1^ Department of Pediatrics, University of Kentucky, Lexington KY, United States; ^2^ Department of Physiology, University of Kentucky, Lexington KY, United States; ^3^ Department of Biostatistics, University of Kentucky, Lexington KY, United States

**Keywords:** neonatal abstinence syndrome (NAS), opioid withdrawal, morphine, clonidine, *in vitro* model, cell death, prefrontal cortex, hippocampus

## Abstract

Neonatal abstinence syndrome (NAS) refers to cadre of withdrawal manifestations in infants born to mothers who used illicit and licit substances during pregnancy. The increasing prevalence of NAS has been largely due to the maternal use of opioids during pregnancy. NAS contributes to increased morbidity and long-term disability in surviving infants. Clinically, oral opioid therapies for opioid exposure have been a standard treatment with morphine (MO) being the most commonly used medication. Recently, a non-opioid agent, clonidine (CD) has also been used with potentially favorable short- and long-term outcomes in infants. However, data regarding the cellular and molecular effects of these treatments on the developing brain is still lacking due to a lack of a reliable animal model that targets the neonatal brain. To address this gap in knowledge we determined the effects of MO or CD on the cell death of neonatal cortical explant cultures that were exposed to oxycodone (OXY) *in utero*. Sprague Dawley rats were randomized and implanted with programmable infusion pumps before mating to receive either the OXY (dose increasing from 1.21–1.90 mg/kg/day to a maximum dose of 2.86–3.49 mg/kg/day) or normal saline (NS) throughout pregnancy and until one week after delivery. Male and female rat pups were sacrificed on postnatal day 4, and the prefrontal cortex (PFC) and hippocampus (HC) were dissected and treated with MO (0.10–1.00 µM) or CD (1.20–120.00 µM) in culture media. After 5 days of treatment the explants were labeled with propidium iodide to detect cell death. Dead cells were analyzed and counted under fluorescence microscopy. In explants from the PFC, cell death was greater in those prenatally exposed to OXY and postnatally treated with MO (OXY/MO) (736.8 ± 76.5) compared to OXY/CD (620.9 ± 75.0; *p* = 0.005). In the HC explants, mean cell death counts were not significantly different between groups regardless of prenatal exposure or postnatal treatment (*p* = 0.19). The PFC is vital in controlling higher-order executive functions such as behavioral flexibility, learning and working memory. Therefore, our finding is consistent with executive function problems in children with prenatal opioid exposure.

## Introduction

Opioid use during pregnancy is reaching record levels in the United States (1, 2). The recent data showed that the incidence of illicit drug use including opioids and marijuana among women of reproductive age was around 16.3% with 5.8% use during pregnancy (3). The increase in maternal opioid use during pregnancy has led to a dramatic increase in Neonatal Abstinence Syndrome (NAS) or more recently termed Neonatal Opioid Withdrawal Syndrome (NOWS) in those infants exposed to opioids *in utero* (4). The incidence of NOWS ranged from 4 to 423 cases (mean 31.8 ± 75.9) per 1,000 birth admissions from the cross-sectional study in the United State from 2016 to 2017 (5). In 2017, the Healthcare Cost and Utilization Project estimated that for every 1,000 newborn hospital stays, 7 were diagnosed with NAS. These babies experience a constellation of symptoms characterized by central nervous system hyperirritability (characterized by incessant and high-pitched cries, tremor), autonomic nervous system dysfunction (temperature instability, nasal stuffiness) and gastrointestinal disturbances (vomiting, diarrhea, poor feeding) (6). Seizures may occur in up to 2%–11% in infants with NAS (7, 8). The current literature supports the use of opioids as a first line pharmacologic treatment in tapering doses for NAS (9). Morphine is the most commonly used medication with small percentages of infants being treated with methadone, and a very small percentage receiving buprenorphine (10). However, it remains unclear how opioid treatment of NAS affects long-term outcomes for these infants. Pre- or perinatal exposure to opioids is associated with long-term effects on neurodevelopment and cognitive functions in children (11–13), decreased brain volumes (14) and lower fractional anisotropy in several areas on the brain magnetic neuroimaging reflecting decreased myelination (15). Preclinical studies also reveal concerning effects of opioid exposure on the developing brain (16) including inhibition of neural progenitor cell differentiation (17), decrease in neurogenesis (18) and impairment of synaptic plasticity (19, 20). Moreover, perinatal exposure to opioids alters the ontogeny of the stress-axis (21, 22) and immune response (23). Therefore, it is important to consider other effective non-opioid treatments for NAS to avoid further exposing the developing brain to opioids and to ultimately improve both short- and long-term clinical outcomes.

Clonidine, an alpha-2 adrenergic agonist that has sedative properties, has been used in animal models of naloxone-induced precipitated withdrawal to ameliorate withdrawal symptoms from adult opioid-addicted rats (24, 25). Clinical studies report that clonidine is an effective treatment for NAS as an adjunct therapy with morphine (26) or chloral hydrate (27). We report that in the pilot clinical study, clonidine treatment is also effective as a monotherapy for NAS and results in improved short-term neurodevelopmental assessment and a shorter length of treatment as compared to morphine treatment (28). The mechanisms whereby neonatal exposure to opioids or clonidine may alter neurological development have not been clearly determined. Virtually no data exist on the molecular and cellular effects underlying the long-term deficits in these children and there are currently only limited animal models to determine such effects.

To begin to address this deficiency in model systems we utilized neonatal explant cultures from animals that were exposed to oxycodone (OXY) *in utero* and determined the effects of postnatal morphine or clonidine exposure on cell death. Organotypic explant cultures have been used extensively to study mechanisms of cell death following neurotoxic insults (29–32). Furthermore, they have advantages over isolated *in vitro* culture systems in that the microenvironment is maintained between neurons and glia, the cultures can be maintained for weeks at a time and they can be pharmacologically manipulated with drug treatments to assess cell death, cell function and gene expression (32). Additionally, the use of an *in vitro* model avoids the complex maternal care and behaviors that can confound *in vivo* models of early brain development (33). The development of a reliable *in vitro* model is critical to understanding the long-term molecular changes that occur in the brain in babies experiencing NAS. We hypothesized that postnatal treatment with clonidine decreased cell death in stress-responsive brain regions including the prefrontal cortex and the hippocampus as compared to the treatment with morphine using an organotypic explant culture model.

## Materials and methods

### Animals and perinatal treatment

The study protocol was approved by the University of Kentucky Institutional Animal Care and Use Committee. Virgin Sprague Dawley rats (Harlan, Indianapolis, IN) weighing 216.5–259 g (mean 242.7 g) (*n* = 10) were housed individually at 22–25 °C and maintained in a 14L:10D photoperiod (lights on at 0500 am) with regulated 30%–70% humidity. Rat chow and water were provided *ad libitum*.

Once released from quarantine, the females were implanted with programmable micro-infusion pumps (iPrecio® Model SMP-200) (iPRECIO®, Alzet, Cupertino, CA) under isoflurane anesthesia. The tips of the tubes were tunneled and positioned for subcutaneous infusion on the nape of the neck. The animals were randomly assigned to receive either oxycodone (OXY) (Mallinckrodt, St. Louis, MO) (100 mg/ml, diluted in normal saline (*n* = 5) or normal saline control (NS) (*n* = 5) on a day of implantation, continued for one week before mating, throughout pregnancy and one week after delivery. In OXY group, the rats received the basal dose of 0.2 *μ*l/hr for one day (OXY dose from basal rate was approximately 1.21–1.9 mg/kg/day), then started to receive escalating doses by pulsatile infusion twice a day during mating and pregnancy. Since the pumps needed to be pre-programmed before implantation, the doses were escalated according to the expected weight gain during pregnancy and the possible development of tolerance to opioid. Each female was housed with the male breeder one week after the implantation of the infusion pumps.

To mimic human use the pulsed dose was escalated as follows. Weeks 1–2: A basal rate of 0.2 *μ*l/hr and a pulse of 1 *μ*l/hr for one hour twice a day was administered for 2 weeks (daily dose from pulse infusion of approximately 0.77–0.87 mg/kg, total daily dose 2.46–2.77 mg/kg/day). Week 3: A basal rate of 0.2 *μ*l/hr and a pulse of 2 *μ*l/hr for one hour twice a day was administered for 1 week (daily dose from pulse infusion of approximately 1.36–1.54 mg/kg, total daily dose 2.85–3.23 mg/kg/day). Week 4: A basal rate of 0.2 *μ*l/hr and a pulse of 3 *μ*l/hr for one hour twice a day was administered for 1 week (dose from pulse infusion of approximately 1.65–2.00 mg/kg, total daily dose 2.86–3.49 mg/kg/day). Finally, Week 5: A basal rate of 0.2 *μ*l/hr and a pulse of 2 *μ*l/hr for one hour twice a day was administered until sacrificed (daily dose from pulse infusion of approximately 1.54 mg/kg, total daily dose 3.23 mg/kg/day). The NS rats group received NS subcutaneously at the same pre-programmed infusion rates.

GD 0 was designated as the day that sperm were detected in the vaginal smear, and the females were individually housed thereafter. On postnatal day (PD) 1, average of 22 days after GD 0, the pups were counted and weighed. The dams were allowed to nurse their own pups while continuing to receive treatment from the infusion pump.

**Figure 1:**
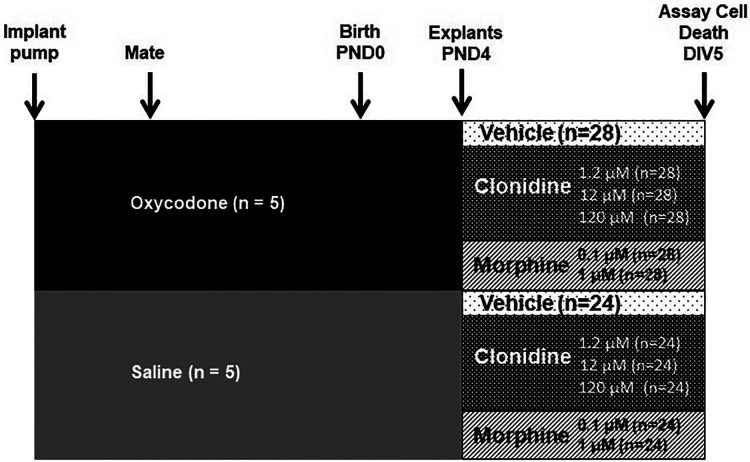
Schematic of pre- and post-natal treatment regimen.

From the 5 dams in prenatal NS group, there were total of 24 pups (12 male and 12 female pups). From the 5 dams in prenatal OXY group, there were total of 28 pups (16 male and 12 female pups). At least one explant from each pup (both PFC and HC) was treated with each one of the six postnatal treatments, the explants were run in duplicate for each treatment. So the n of the pups for prenatal NS (control) for all postnatal treatments = 24 (combined male and female), and n for prenatal oxycodone for all postnatal treatments = 28 (combined male and female), Figure 1.

### Organotypic cortical explants

See Figure 1. Cortical explants are isolated from PND 3–4 rat pups, as previous described (29, 34, 35) with slight modifications. PD 3–4 is chosen because it is the optimal age of development for them to survive, but still can differentiate adequately and can potentially harvest both the PFC and HC from the same animal. Additionally, cutting the explants from younger animals is technically challenging. Pups were sexed and brains were isolated and sectioned, 300 µm, in cold dissection media containing Gey's balanced salt solution (G9779, Sigma-Aldrich, Saint Louis, MO), 0.2 M MgCl2 and 37.5% glucose on a vibratome from Bregma −.36 to −2.64 mm. Approximately 8–10 slices were harvested per brain. In cold dissection media plus ketamine HCl (Ketaset, NLS Animal Heath). Each brain was isolated for the prefrontal cortex and for the hippocampus. Individual cortices were plated on Millicell-CM membranes (PICMO3050, Fisher, Hampton, NH) in wells containing 1X Basal Medium Eagle (B9638, Sigma-Aldrich), Hanks' Balanced Salt Solution (14,025, Invitrogen), heat-inactivated horse serum (3H30074.03, Fisher), 37.5% glucose in Geys BSS, glutamax (35,050, Invitrogen, Carlsbad, CA), and penicillin/streptomycin (15,140, Invitrogen). Explants remained in culture at 34 °C with 5% CO_2_. Media was changed every three days. Healthy explants are transparent with smooth edges while overfed explants become opaque and underfed explants thin to the point that they are undetectable (35). After 3 days on the culture media, the explants were treated with one these 6 treatments: vehicle either alone (CON), or with 0.1 or 1 µM morphine (MO), or 1.2, 12 or 120 µM clonidine (CD). These concentrations of morphine treatment were used to cover the range of the mean plasma concentrations of 125 up to above 300 and 167 ± 77 ng/ml that were reported in the neonates that received the therapeutic doses of morphine (36, 37). These concentrations of clonidine were used to cover the extrapolated intra-cerebroventricular concentration reported to prevent the reduction in the hypothalamic noradrenaline after naloxone-induced withdrawal in chronically morphine treated rats (38).

### Assessment of cell death

After 5 days of treatment, explants were washed with 0.1 M PBS and incubated with 5 µg/ml of propidium iodide (PI) (1 mg/ml in H2O, P4170, Sigma-Aldrich) in BME for 30 min. Explants were washed (0.1M PBS) and visualized using a fluorescent microscope. PI entered cells that had a porous cell membranes, indicating damage, and bound to DNA. PI uptake indicated cell death and fluoresced red (emission at 630 nm) under green light (excited at 495 nm). Pictures, 20X magnification of explants, were captured using an image capture program, SPOT Advanced. Red (dead) cells per frame were then counted using a Nikon NIS-Element software®. Pictures were coded and analyzed blindly.

### Statistical analysis

Linear mixed effects models with a random effect for litter were used for statistical analyses with statistical significance defined as *p* < 0.05. Because there were no significant dose effects in MO or CD treatment groups, the results from the 2 MO concentrations (0.1 or 1 µM) and the 3 CD (1.2, 12 or 120 µM) groups were combined for further analysis. The interactions between gender and treatment groups were not significant; therefore, the results from both male and female offspring were combined.

## Results

### In the PFC

**Figure 2:**
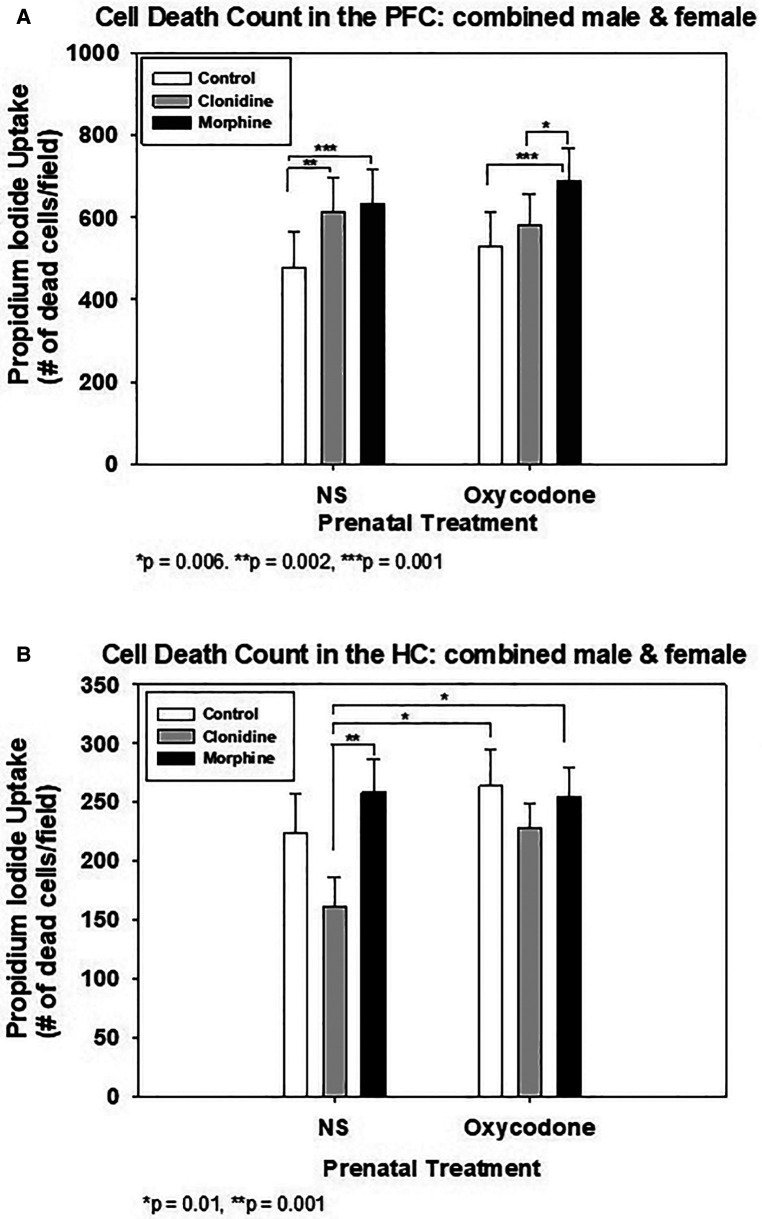
Quantification of cell death in organotypic explants following treatment with clonidine or morphine. (**A**) Prefrontal Cortex (PFC) Explants. (**B**) Hippocampus (HC) explants.

Figure 2A shows the cell death counts with MO or CD treatment in the PFC explants. In explants from prenatally exposed OXY pups, only postnatal treatment with morphine, not clonidine, increased cell death compared to CON. Postnatal morphine also increased cell death compared to clonidine. In explants from prenatally exposed normal saline (NS) pups, both morphine and clonidine increased cell death compared to CON.

### In the hippocampus

Figure 2B shows the effect of MO or CD on the HC explants from prenatal exposure to either OXY or NS. In either prenatal OXY or NS groups, postnatal treatment with MO or CD had no effect on cell death when compared to CON. However, in the prenatal NS explants, postnatal CD treatment decreased cell death compared to MO. Postnatal treatment with CD also decreased cell death in the prenatal NS explants when compared to either postnatal treatment with MO or CON in prenatal OXY explants. The decline in cell death in the prenatal NS-postnatal CD group did not differ from the prenatal NS –postnatal CON group.

**Figure 3:**
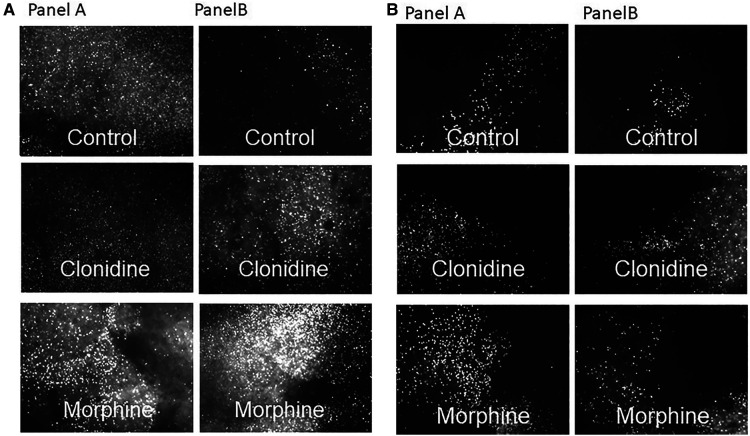
Propidium iodide staining for cell death from the PFC (**A**) and hippocampus (**B**) under fluorescence microscopy, explants were prenatally exposed to NS (panel A) and to OXY (panel B) with postnatal treatment with control, clonidine and morphine respectively.

Assessment of cell death by propidium iodide staining under fluorescence microscopy were as shown, from the PFC (Figure 3A) and from the hippocampus (Figure 3B). Worst staining for cell death noted in postnatal MO treatment group, see Figure 2 for cell death count.

## Discussion

To the best of our knowledge this is the first study to use an invitro model of organotypic cortical explants to study the effects of non-opioid vs. opioid treatment for NAS/ NOWS on cell death. Our results showed the anatomical site- and prenatal exposure- specific protective effects of clonidine on cell death. Although organotypic explant cultures have been used to study the effects of certain treatments on neuronal toxicity and cell death (31, 39), they have not been previously used to study the effects of prenatal opioid exposure and postnatal treatment for NAS.

From this pilot study, postnatal exposure to clonidine decreased cell death in the prefrontal cortex cortical explants compared to postnatal exposure to morphine when the rats were prenatally exposed to oxycodone; this possible protective effect was not noted when the rats were prenatally exposed to NS. In the hippocampus, clonidine also decreased cell death compared to morphine, when rats were prenatally exposed to NS. However, this effect was not present when the rats were prenatally exposed to oxycodone. Postnatal clonidine also decreased cell death in the hippocampus of prenatal NS rats when compared to postnatal treatment with morphine or control in prenatal oxycodone group.

As hypothesized, our results suggested that when the animals were prenatally exposed to opioid, postnatal treatment with the non-opioid clonidine led to a decrease in cell death in the prefrontal cortex as compared to treatment with morphine. This finding supports the possibility of using a non-opioid therapeutic agent as an alternative or adjunctive therapy for NAS/ NOWS as a growing body of evidence have suggested adverse effects of opioids on the developing brain. Our results support the findings from the previous preclinical study by Bajic et al. that morphine exposure during the neonatal period (PD1–7) increased the density of neuronal cell death in the neonatal rat cortex and amygdala (40). Others also reported increased neuronal cell death (41) and reduced cortical thickness and the numbers of neurons in the fetal frontal cerebral cortex in the offspring (42) after prolonged intrauterine morphine exposure. Although we did not explore the mechanisms of cell death in this study, one of the mechanisms by which morphine enhances neuronal cell death is reported to be increased apoptosis *via* a caspase-3 dependent pathway (40, 43, 44). Oxidative stress has been described as another cellular mechanism involved in opioid neurotoxicity (45). We found brain region-specific effects of the pre- and postnatal opioid treatment on cell death in this study. Our findings are in line with Bajic et al. that the prefrontal cortex but not the hippocampus, is one of the supra-spinal regions susceptible to opioid toxicity. This may be due to the relative densities of glutamatergic neurons which may lead to increased neurotoxicity as has been shown in other paradigms (46). A limitation of our study is that we did not perform immunohistochemistry to identify the specific cell types of dead cells. However, previous studies showed that opioids disrupt neuronal and glial maturation by context-dependent, modulatory effects throughout ontogeny (47). Future study should aim to identify cell types and possible mechanisms underlying our findings. There seemed to be significant amount of background, which can be potentially explained by the thickness of the samples. All explants do not thin at the same rate. Another limitation is that we used propidium iodide staining which only crosses the membranes of the dead cells and detects both apoptotic and necrotic cell death. Annexin V technic should be considered for future experiments to specifically assess apoptotic cell death (48). Of note, there were significant amount of cell death in the explants from NS exposed rat pups suggesting that this may be part of a normal process or may reflect what happens to the cells in the explant cultures.

Clonidine, on the other hand, has been reported to provide dose and brain region-specific neuroprotective effects for cerebral ischemia in the *in vivo* model (49, 50). In vitro, clonidine decreases the neuronal cell injury caused by N-methyl-d-aspartate (NMDA) receptor agonist exposure, an effect which is abolished by the selective alpha2-adrenoceptor antagonist yohimbine in primary cortical neuron cultures (51). The mechanism by which clonidine may have less toxic effects as a treatment for neonatal drug withdrawal requires further elucidation. In addition to preventing the elevation of norepinephrine, thereby ameliorating sympathetic hyperactivity in NAS (52) which in turn can alleviate withdrawal symptoms, clonidine may provide neuroprotection by reducing the release of glutamate resulting in decreased NMDA activation and neuronal damage (53). Further studies are needed to explore the mechanisms by which clonidine may provide neuroprotective effects after perinatal opioid exposure.

Interestingly, the treatment group with highest cell death in the PFC was among the pups that were prenatally exposed to OXY and treated postnatally with morphine. The significance of this finding may be related to the fetal programming by prenatal opioid exposure (54). This concept was grounded on the pathophysiology of the effects of prenatal cocaine exposure (55) and early life stress (56), wherein prenatal exposure to stress or substances of abuse can potentially lead to altered programming of brain development and adverse short- and long-term neurodevelopmental outcomes. Fetal programming involves the processes by which conditions during critical periods of cellular proliferation, differentiation, and maturation affect the developing brain and how the brain responds to and interacts with these conditions, which in turn can affect cell survival (57). Besides the effects on the stress-axis (58), prenatal stress (59) and opioids (60, 61) can alter the availability of neurotrophic factors such as brain-derived neurotrophic factor (BDNF) which may be one of the key signaling pathways that alters cell survival. Further study including the use of animal models is required to elucidate how prenatal opioid exposure can possibly make the brain more susceptible to a postnatal averse environment and investigate its effects on the long term outcomes (62).

We did not find significant interactions between treatment groups and gender in this study which could be due to our small sample size, therefore the results were combined. However, previous studies have described the gender-specific susceptibilities or vulnerabilities that impact cognitive, executive and behavioral outcomes after prenatal substance exposure (63). Our group previously reported more notable hyperactivity in the open field test in prenatal oxycodone-exposed male offspring compared to females (64). Prenatal opioid exposure is consistently associated with behavioral issues, primarily with the symptoms of attention–deficit hyperactivity disorder (ADHD) in children (65, 66). Behavior and attention is significantly regulated by the PFC; weaker structure and function of the PFC is associated with attention deficit/ hyperactivity (67). There exists emerging evidence that the corticolimbic system undergoes age and gender –specific development (68). Altogether, more studies are needed to verify the effects of perinatal opioid exposure/ treatment on the development on the corticolimbic system,interaction with genders and other potential postnatal interventions that may improve the long term outcomes (69, 70).

## Conclusions

In this pilot study we attempted to develop an *in vitro* model to study the effects of opioid (morphine) vs. non-opioid (clonidine) treatment for NAS/NOWS after prenatal exposure to oxycodone on cell death by using organotypic explant cultures from two of the corticolimbic- regions, the prefrontal cortex and the hippocampus. We found that post-natal treatment with clonidine may have effects to decrease cell deaths in the PFC as compared to morphine treatment, a result which supports consideration to use clonidine as another option for NAS/NOWS treatment. No differences in the effects of postnatal treatment on cell death were found in the hippocampus when prenatally exposed to oxycodone, but in prenatal NS-treated explants, postnatal clonidine treatment also decreased cell death compared to morphine. Although our experiments showed interesting findings from the small sample sizes, future studies are warranted as there are certain limitations. Those studies may utilize this model to investigate other pharmacologic treatment choices for NAS/NOWS and further determine the mechanisms for cell death/ cell survival and other pathophysiology by which prenatal opioid exposure and postnatal treatment may affect brain development.

## Data Availability

The original contributions presented in the study are included in the article/Supplementary Material, further inquiries can be directed to the corresponding author/s.
